# Association of eosinopenia with worsening prognosis in hospitalized Azvudine-treated COVID-19 patients: a retrospective cohort study

**DOI:** 10.3389/fimmu.2023.1320973

**Published:** 2023-12-15

**Authors:** Xiaomin Wang, Yating Dian, Qian Zhou, Guangtong Deng, Rui Wei, Furong Zeng

**Affiliations:** ^1^ Department of Oncology, Xiangya Hospital, Central South University, Changsha, Hunan, China; ^2^ Department of Dermatology, Xiangya Hospital, Central South University, Changsha, China; ^3^ National Engineering Research Center of Personalized Diagnostic and Therapeutic Technology, Changsha, Hunan, China; ^4^ Furong Laboratory, Changsha, Hunan, China; ^5^ Hunan Key Laboratory of Skin Cancer and Psoriasis, Hunan Engineering Research Center of Skin Health and Disease, Xiangya Hospital, Central South University, Changsha, China; ^6^ National Clinical Research Center for Geriatric Disorders, Xiangya Hospital, Changsha, China; ^7^ Tianjin Institute of Urology, The Second Hospital of Tianjin Medical University, Tianjin, China

**Keywords:** COVID-19, eosinopenia, azvudine, prognosis, outcome

## Abstract

**Background:**

Current guidelines prioritize the use of Azvudine in Coronavirus Disease 2019 (COVID-19) patients, while biomarkers for prognosis in Azvudine-treated COVID-19 patients are still lacking. Here, we aim to assess the prognostic value of eosinopenia in Azvudine-treated COVID-19 patients.

**Methods:**

We retrospectively reviewed 290 consecutive Azvudine-treated hospitalized COVID-19 patients. Clinical characteristics and prognosis data were analyzed between patients with eosinopenia and with normal eosinophil levels.

**Results:**

A total of 290 patients were enrolled in this study, with a median age of 69 years. Among them, 40.69% presented with eosinopenia and 59.31% had normal eosinophil levels. Common symptoms included cough (87.6%), expectoration (76.2%), fever (67.9%), poor appetite (47.2%), and polypnea (46.6%). Compared to patients with normal eosinophil levels, those with eosinopenia were older and less likely to experience fatigue (25.4% vs. 39.0%, P=0.016). Significant differences in laboratory parameters, particularly in blood routine and blood biochemical indicators, were observed between the two groups. Patients with eosinopenia were also less likely to develop severe illness subtypes, requiring more medication and oxygen support. The Cox proportional hazard model showed that eosinopenia was associated with worsening progression in Azvudine-treated COVID-19 patients (adjusted hazard ratio=2.79, 95% confidence interval: 1.04, 7.50), adjusting for potential confounders.

**Conclusion:**

Eosinopenia is associated with worsening prognosis in Azvudine-treated COVID-19 patients.

## 1 Introduction

The coronavirus disease 2019 (COVID-19) pandemic emerged as a global public health crisis, leading to significant morbidity and mortality worldwide (1). Azvudine, the first homegrown anti-COVID-19 drug in China, has been authorized for the treatment of COVID-19 patients (2). In a phase 3 multicenter clinical study, Azvudine significantly improved clinical symptoms of COVID-19 patients (40.43% vs. 10.87%) and achieved superior clinical outcomes (3). However, not all COVID-19 patients derive the same benefits from Azvudine treatment, necessitating the urgent exploration of prognostic biomarkers in Azvudine-treated COVID-19 patients.

Eosinophils, a type of white blood cell involved in allergic reactions and antiviral defense, have been described as an indirect indicator of infection in previous studies (4, 5). Recent studies have supported the potential diagnostic value of eosinopenia in COVID-19 and explored the prognostic significance of eosinophils in COVID-19 patients (6–8). A retrospective study by Zein et al. found that COVID-19 patients treated with inhaled corticosteroids and exhibiting baseline eosinophilia had better outcomes (9). Interestingly, existing evidence suggests that patients with longstanding eosinophil-associated complications are not at an increased risk of severe disease (10). These studies have established an association between eosinophil levels and COVID-19 diagnosis and outcomes. However, the real-world evidence regarding the association between eosinopenia and prognosis in Azvudine-treated COVID-19 patients remains unclear.

Herein, the present study analyzes data from a hospital to assess the prognostic value of eosinopenia in Azvudine-treated COVID-19 patients.

## 2 Methods

### 2.1 Study design and patients

This single-center, retrospective study was conducted following the Declaration of Helsinki. The study was approved by the institutional ethics board of the hospital (No. 202002024), and the requirement for informed consent in this retrospective cohort was waived. Consecutive COVID-19 patients admitted to our hospital were enrolled from December 5, 2022, to January 31, 2023. The study included hospitalized patients with a confirmed diagnosis of SARS-CoV-2 infection who received Azvudine treatment. The patients with the following conditions were excluded: 1) younger than 18 years or received oxygen support on admission; 2) received any other antiviral therapy rather than Azvudine; 3) without regular blood tests; 4) with eosinophilia or received any drugs that affect eosinophil level.

### 2.2 Data extraction

The demographic characteristics (age and sex), and clinical data (symptoms, comorbid conditions, laboratory findings, treatments, and outcomes) of patients on admissions were retrieved from electronic medical records by two investigators. The health records were then linked with anonymized vaccination records provided by the Department of Immunization, Center for Disease Control and Prevention of Hunan Province using unique identification numbers (China Identity Card) (11).

### 2.3 Definition of conditions

We defined eosinopenia based on previous reports (12). The definition of severe COVID-19 patients in this study was based on specific criteria, including a respiratory rate of ≥30 breaths per minute, oxygen saturation ≤93%, a ratio of partial pressure of arterial oxygen to fraction of inspired oxygen (PaO2/FiO2) ≤300 mmHg, or lung infiltrates >50% on admission (13).

### 2.4 Outcomes

The outcome in this study is a composite outcome of disease progression including all-cause death, intensive care unit admission, and oxygen therapy.

### 2.5 Statistical analysis

The Chi-square test or Fisher’s exact test was performed to compare categorical variables between the eosinopenia and normal groups. Continuous variables were compared using the Mann-Whitney U test and were expressed as median (interquartile range [IQR]) values. Survival curves were estimated using the Kaplan-Meier method and a two-sided log-rank test stratified by study was used to compare results between groups. The hazard ratios (HR) and 95% confidence intervals (CI) for composite outcome were estimated using Cox regression analysis, and then potential confounders were adjusted. For all the analyses, *P*<0.05 was considered statistically significant. Statistical analyses were performed using R software (version 4.2.1) and SPSS software (version 26.0).

## 3 Results

### 3.1 Baseline characteristics of the patients of COVID-19

**Figure 1:**
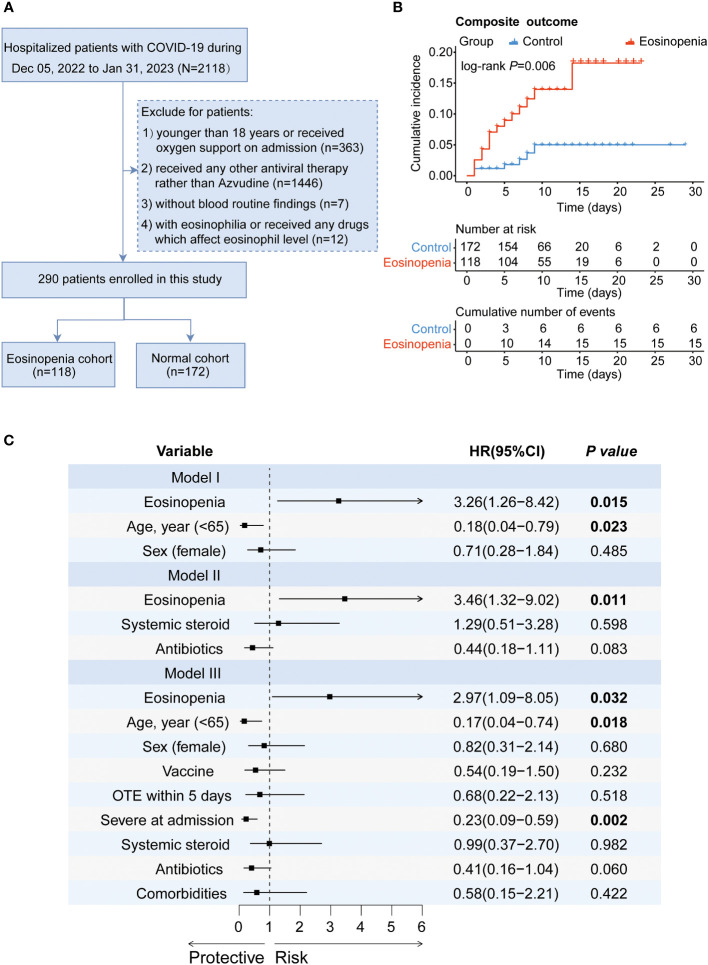
The effect of eosinopenia on the outcome of patients treated with Azvudine. **(A)** Flowchart of patient recruitment. **(B)** Kaplan-Meier curve of cumulative incidence of composite outcome for Azvudine-treated COVID-19 patients with eosinopenia versus patients with normal eosinophil. **(C)** Forest plot of the results of Cox regression analysis in different models. Model I adjusted for age and sex. Model II adjusted for the use of systemic steroid and antibiotics. Model III adjusted age, sex, vaccine, OTE, severity, the use of systemic steroid and antibiotics, and comorbidities. HR, hazard ratio; CI, confidence interval; OTE, time from symptom onset to treatment exposure.

**Figure 1** presents the flowchart for patient recruitment. Initially, 2118 patients were screened from the medical record system between December 05, 2022 and January 31, 2023. Among them, a total of 290 Azvudine-treated COVID-19 patients without oxygen therapy upon admission met the eligibility criteria for inclusion. Of these patients, 118 (40.69%) had eosinopenia and 172 (59.31%) had normal eosinophil levels. **Table 1** provides an overview of the baseline characteristics of these individuals. Among the 290 enrolled patients, the median age was 69 years (IQR [59, 78]; range, 18–95 years), with an age range of 18 to 95 years, and 178 (61.4%) were male. The five most common symptoms reported at the onset of illness were cough (254 [87.6%]), expectoration (221 [76.2%]), fever (197 [67.9%]), poor appetite (137 [47.2%]), and polypnea (135 [46.6%]). Compared to patients with normal eosinophil levels, patients with eosinopenia were older (median age [IQR]: 72 [59, 81] years vs. 67 [58, 75] years; P=0.04) and less likely to experience fatigue (25.4% vs. 39.0%, P=0.016). No significant differences were observed between the eosinopenia and normal eosinophil groups regarding other symptoms. Among the enrolled patients, 239 (82.4%) had other preexisting chronic complications including hypertension (44.5%), coronary heart disease (24.5%), diabetes mellitus (24.1%), and chronic obstructive pulmonary disease (COPD, 5.5%). Patients with eosinopenia were more likely to have COPD compared to those without eosinopenia (9.3% vs. 2.9%, P=0.019). No significant differences were found between the eosinopenia and normal eosinophil groups in terms of other preexisting comorbidities.

**Table 1:** Characteristics of the patients with COVID-19.

Characteristics	Total	Eosinopenia	Normal	*P* value
	(N=290)	(n=118)	(n=172)	
Demographic characteristics				
Age, median (IQR)	69 (59, 78)	72 (59, 81)	67 (58, 75)	**0.04**
Sex, n (%)				0.528
Male	178 (61.4)	75 (63.6)	103 (59.9)	
Female	112 (38.6)	43 (36.4)	69 (40.1)	
Symptoms, n (%)				
Cough	254 (87.6)	101 (85.6)	153 (89.0)	0.394
Expectoration	221 (76.2)	92 (78.0)	129 (75.0)	0.56
Fever	197 (67.9)	82 (69.5)	115 (66.9)	0.637
Poor Appetite	137 (47.2)	60 (50.8)	77 (44.8)	0.308
Polypnea	135 (46.6)	61 (51.7)	74 (43.0)	0.146
Fatigue	97 (33.4)	30 (25.4)	67 (39.0)	**0.016**
Stuffiness	84 (29.0)	31 (26.3)	53 (30.8)	0.402
Preexisting condition, n (%)				
Hypertension	129 (44.5)	63 (53.4)	66 (38.4)	**0.011**
Coronary heart disease	71 (24.5)	36 (30.5)	35 (20.3)	0.048
Diabetes mellitus	70 (24.1)	28 (23.7)	42 (24.4)	0.893
COPD	16 (5.5)	11 (9.3)	5 (2.9)	**0.019**
Cancer	27 (9.3)	12 (10.2)	15 (8.7)	0.677

IQR, interquartile range; COPD, chronic obstructive pulmonary disease. Bold values means highlight P < 0.05.

### 3.2 Laboratory findings in patients of COVID-19 on admission

**Table 2** displays the laboratory findings on admission for COVID-19 patients with eosinopenia and those with normal eosinophil levels. Significant variations in laboratory test results were observed between the two groups. Blood routine tests revealed a common decrease in eosinophil count among these patients (40.69%). COVID-19 patients with eosinopenia exhibited a lower median lymphocyte count (median: 0.7 [IQR: 0.5, 1.1] ×10^9^/L) compared to patients with normal eosinophil levels (median: 1.1 [IQR: 0.8, 1.4] ×109/L, P<0.001). Interestingly, patients with eosinopenia had a higher median neutrophil count (median: 4.5 [IQR: 2.97, 6.9] ×10^9^/L) than those with normal eosinophil levels (median: 3.6 [IQR: 2.72, 4.88] ×10^9^/L, P=0.001). Consequently, the neutrophil-to-lymphocytes ratio (NLR) was significantly higher in patients with eosinopenia compared to those with normal eosinophil levels (median: 6.55 [IQR: 3.40, 9.89] vs. median: 3.16 [IQR: 2.19, 5.12], P<0.001).

**Table 2:** Laboratory findings of COVID-19 patients.

Characteristics	Total	Eosinopenia	Normal	*P* value
	(N=290)	(n=118)	(n=172)	
Blood routine, median (IQR)				
WBC (×10^9^/L)	5.5 (4.5, 7.43)	5.85 (4.5, 8.23)	5.5 (4.43, 7.28)	0.353
Lymphocyte (×10^9^/L)	1 (0.7, 1.3)	0.7 (0.5, 1.1)	1.1 (0.8, 1.4)	**<0.001**
Neutrophil (×10^9^/L)	3.9 (2.8, 5.7)	4.5 (2.97, 6.9)	3.6 (2.72, 4.88)	**0.001**
NLR	3.90 (2.44,7.44)	6.55 (3.40,9.89)	3.16 (2.19,5.12)	**<0.001**
Blood biochemical, median (IQR)				
Tbil (μmol/L)	9.9 (7.43, 13)	9.85 (7.1, 13.02)	9.9 (7.77, 13)	0.713
Dbil (μmol/L)	3.55 (2.7, 4.68)	3.6 (2.6, 5.2)	3.5 (2.8, 4.4)	0.717
Albumin (g/L)	34.5 (31.93, 37.3)	33.65 (30.35, 36.5)	35.05 (32.97, 37.8)	**0.001**
Globulin (g/L)	27.85 (25.3, 31.1)	28.05 (24.1, 31.9)	27.7 (25.57, 30.55)	0.845
ALT (U/L)	24.1 (15.9, 38.4)	24.7 (17.9, 40.43)	23.3 (15.45, 36.25)	0.165
AST (U/L)	26.1 (19.6, 38.4)	29.5 (21.37, 46.47)	23.6 (18.75, 34.4)	**0.001**
Scr (μmol/L)	69.75 (59, 85.4)	74.3 (59.7, 92)	66.4 (57.6, 80)	**0.007**
BUN (mmol/L)	5.46 (4.13, 7.56)	6.47 (4.85, 8.71)	4.95 (3.95, 6.45)	**<0.001**
LDH (U/L)	215.8 (182, 263.1)	239 (205, 306)	206.4 (173.85, 241)	**<0.001**
CK (U/L)	59.4 (38.3, 96)	69.1 (41.2, 125.5)	53.05 (36.97, 82.87)	**0.004**
CK-MB (U/L)	10.4 (7.8, 13.1)	11.6 (8.9, 14.6)	9.65 (7.4, 11.7)	**0.001**
Myoglobin (μg/L)	58.5 (40.4, 91.6)	77 (49.8, 150.7)	49.85 (36.33, 73.25)	**<0.001**
TnI (ug/mL)	0.04 (0.02, 0.13)	0.06 (0.03, 0.2)	0.03 (0.01, 0.1)	**0.013**
BNP (pg/mL)	251.1 (126.6, 719.8)	496.68 (165.1, 1373.7)	180.87 (121.1, 415.4)	**<0.001**
HbA1c (%)	6.2 (5.9, 6.9)	6.1 (5.6, 6.55)	6.55 (5.97, 7.43)	**0.011**
Lactic (mmol/L)	1.41 (1.04, 1.87)	1.46 (1.01, 2.06)	1.41 (1, 1.79)	0.454
PCT (ng/mL)	0.05 (0.05, 0.08)	0.06 (0.05, 0.13)	0.05 (0.04, 0.06)	**0.001**
CRP (mg/L)	22.52 (6.4, 75.39)	38.79 (8.34, 79.6)	19.2 (4.76, 62.3)	**0.028**
ESR (mm/h)	51.5 (35, 71.25)	50 (33.25, 71.25)	53.5 (36.25, 72.5)	0.384
Ferritin (ng/mL)	795.8 (437.9, 1086.5)	853.3 (503.05, 1172)	774.6 (401, 1062)	0.325
Coagulation tests, median (IQR)				
D-Dimer (mg/L)	0.18 (0.09, 0.36)	0.22 (0.11, 0.54)	0.16 (0.08, 0.28)	**0.01**
FIB (g/L)	4.37 (3.39, 5.22)	4.39 (3.54, 5.21)	4.26 (3.3, 5.33)	0.409
Inflammatory factors, median (IQR)				
TNF-a (pg/mL)	8.72 (3.91, 13.1)	11.1 (5.42, 14.75)	8.24 (2.91, 11.93)	0.067
IL2 (pg/mL)	1.83 (1.32, 2.76)	1.67 (1.27, 2.07)	2.11 (1.3, 3.37)	0.305
IL6 (pg/mL)	8.57 (2.83, 19.72)	9.96 (2.3, 22.02)	8.48 (3.09, 16.53)	0.918
IL10 (pg/mL)	5 (2.97, 5)	5 (4.92, 5.76)	5 (2.25, 5)	**0.025**

IQR, interquartile range; WBC, white blood cell; NLR, neutrophil-to-lymphocyte ratio; TBil, total bilirubin; DBil, direct bilirubin; ALT, alanine aminotransferase; AST, glutamic oxaloacetic transaminase; Scr, serum creatinine; BUN, blood urea nitrogen; LDH, lactate dehydrogenase; CK, creatine kinase; CK-MB, creatine kinase MB; TnI, troponin I; BNP, brain natriuretic peptide precursor; PCT, procalcitonin; CRP, C-reactive protein; ESR, erythrocyte sedimentation rate; FIB, fibrinogen. Bold values means highlight P < 0.05.

Regarding blood biochemical findings, COVID-19 patients with eosinopenia exhibited higher levels of glutamic oxaloacetic transaminase (median: 29.5 [IQR: 21.37, 46.47] U/L vs. median: 23.6 [IQR: 18.75, 34.4] U/L, P=0.001) (**Figure S1A**), serum creatinine (median: 74.3 [IQR: 59.7, 92] μmol/L vs. median: 66.4 [IQR: 57.6, 80] μmol/L, P=0.007), blood urea nitrogen (median: 6.47 [IQR: 4.85, 8.71] mmol/L vs. median: 4.95 [IQR: 3.95, 6.45] mmol/L, P<0.001), lactate dehydrogenase (median: 239 [IQR: 205, 306] U/L vs. median: 206.4 [IQR: 173.85, 241] U/L, P<0.001), creatine kinase (median: 69.1 [IQR: 41.2, 125.5] U/L vs. median: 53.05 [IQR: 36.97, 82.87] U/L, P=0.004), creatine kinase MB (median: 11.6 [IQR: 8.9, 14.6] U/L vs. median: 9.65 [IQR: 7.4, 11.7] U/L, P=0.001), myoglobin (median: 77 [IQR: 49.8, 150.7] μg/L vs. median: 49.85 [IQR: 36.33, 73.25] μg/L, P<0.001), troponin I (median: 0.06 [IQR: 0.03, 0.2] μg/mL vs. median: 0.03 [IQR: 0.01, 0.1] μg/mL, P=0.013), brain natriuretic peptide precursor (BNP, median: 496.68 [IQR: 165.14, 1373.66] pg/mL vs. median: 180.87 [IQR: 121.09, 415.43] pg/mL, P<0.001), procalcitonin (median: 0.06 [IQR: 0.05, 0.13] ng/mL vs. median: 0.05 [IQR: 0.04, 0.06] ng/mL, P=0.001), C-reactive protein (median: 38.79 [IQR: 8.34, 79.6] mg/L vs. median: 19.2 [IQR: 4.76, 62.3] mg/L, P=0.028) (**Figure S1B**), and albumin (median: 33.65 [IQR: 30.35, 36.5] g/L vs. median: 35.05 [IQR: 32.97, 37.8] g/L, P=0.001). In terms of coagulation function markers, patients with eosinopenia had higher levels of D-Dimer (median: 0.22 [IQR: 0.11, 0.54] mg/L vs. median: 0.16 [IQR: 0.08, 0.28] mg/L, P=0.01) (**Figure S1C**) than patients with normal eosinophil levels. Additionally, patients with eosinopenia had higher levels of IL-10 (pg/mL) (median: 5 [IQR: 4.92, 5.76] pg/mL vs. median: 5 [IQR: 2.25, 5] pg/mL, P=0.025) (**Figure S1D**) compared to those with normal eosinophil levels. No significant differences were observed between the two groups for other laboratory findings.

### 3.3 Analysis of associations of eosinopenia with clinical outcomes in Azvudine-treated COVID-19 patients

Subsequently, we compared the severity, treatment, and short-term prognosis of COVID-19 patients in the two groups, as presented in **Table 3**. A total of 185 patients (63.8%) developed severe illness subtypes, and 264 patients (91%) received oxygen therapy. The percentages of patients using nasal cannula oxygen, mask oxygen, high-flow oxygen, and invasive mechanical ventilation were 89.7% (260 patients), 2.8% (8 patients), 4.1% (12 patients), and 3% (1 patient), respectively. Compared to COVID-19 patients with normal eosinophil levels, patients with eosinopenia were less likely to develop severe illness subtypes (55.9% vs. 69.2%, P=0.021). Additionally, patients with eosinopenia received a higher percentage of medications (94.1% vs. 83.1%, P=0.006), including immunoregulators (29.7% vs. 18.6%, P=0.028) and corticosteroids (65.3% vs. 44.2%, P<0.001). Moreover, patients with eosinopenia required a higher level of oxygen support (96.6% vs. 87.2%, P=0.006), including nasal cannula oxygen (94.1% vs. 86.6%, P=0.041) and high-flow oxygen (9.3% vs. 0.36%, P<0.001). During the follow-up period, as of January 31, 2023, three patients (1%) had died, and 284 patients (97.9%) had been discharged, and the rest (3 [1%]) remained hospitalized. Patients with eosinopenia exhibited a higher fatality rate compared to those with normal eosinophil levels (2.5% vs. 0%).

**Table 3:** Disease severity, treatment, and prognosis of COVID-19 patients.

Characteristics	Total	Eosinopenia	Normal	*P* value
	(N=290)	(n=118)	(n=172)	
**Blood routine, median (IQR)**				**0.021**
Mild to moderate	105 (36.2)	52 (44.1)	53 (30.8)	
Severe	185 (63.8)	66 (55.9)	119 (69.2)	
Pharmacotherapy, n (%)				
Immunomodulator	67 (23.1)	35 (29.7)	32 (18.6)	**0.028**
Corticosteroid	153 (52.8)	77 (65.3)	76 (44.2)	**<0.001**
Antibiotic	224 (77.2)	96 (81.4)	128 (74.4)	0.166
Oxygen support, n (%)				
Nasal cannula	260 (89.7)	111 (94.1)	149 (86.6)	**0.041**
Mask oxygen	8 (2.8)	5 (4.2)	3 (1.7)	0.277*
High-flow oxygen	12 (4.1)	11 (9.3)	1 (0.6)	<0.001*
IMV	3 (1)	2 (1.7)	1 (0.6)	0.569*
**Clinical outcome, n (%)**				0.033*
Discharged	284 (97.9)	115 (97.5)	169 (98.3)	
Remained in hospital	3 (1)	0 (0)	3 (1.7)	
Died	3 (1)	3 (2.5)	0 (0)	

*Fisher’s exact test.

IQR, interquartile range; IMV, Invasive mechanical ventilation. Bold values means highlight P < 0.05.

To further assess the association of eosinopenia with the clinical outcome of Azvudine-treated COVID-19 patients, we performed a Kaplan-Meier curve analysis for prognosis. The results suggested that patients with eosinopenia had a worse clinical outcome than those with normal eosinophil levels (P=0.006) (**Figure 1B**). Additionally, a Cox proportional hazard model was performed. Eosinopenia was identified as an important risk factor for prognosis in COVID-19 patients, even after adjusting for age and sex (adjusted hazard ratio [AHR]=3.26, 95% confidence interval [CI]: 1.26-8.42, P=0.015) and further adjusting for immunomodulators, corticosteroids, and antibiotic usage (AHR=3.46, 95% CI: 1.32-9.02, P=0.011), or additionally adjusting for age, sex, severity, time from symptom onset to treatment exposure, preexisting conditions, corticosteroid or antibiotic usage, and vaccination status (AHR=2.98; 95% CI: 1.09-8.05; P=0.032) (**Figure 1C**).

## 4 Discussion

The COVID-19 outbreak has emerged as an ongoing global health crisis (14). Various risk factors have been identified that are associated with disease severity and prognosis in COVID-19 patients (15). Recent studies have shown that eosinopenia is a risk factor for COVID-19 patients, which may aid in disease diagnosis and impact prognosis (7). However, there is limited real-world evidence regarding the prognostic value of eosinopenia in Azvudine-treated COVID-19 patients.

In this study, a total of 290 hospitalized COVID-19 patients were included for final analysis. Our analyses demonstrated a significant association between eosinopenia and an increased risk of composite outcome compared to patients with normal eosinophil levels. These findings indicate that eosinopenia is an important prognostic factor in COVID-19. To the best of our knowledge, this is the first real-world study to explore the prognostic value of eosinopenia in hospitalized Azvudine-treated COVID-19 patients during the pandemic wave in China.

Zhang et al. reported that 53% of COVID-19 individuals had eosinopenia on the day of hospital admission (16). In our study, a slightly lower proportion of Azvudine-treated COVID-19 patients (40.69%) presented with eosinopenia. Notably, some reports have documented eosinopenia in patients presenting with moderate-to-severe COVID-19 (17). However, our results suggest that patients with eosinopenia were less likely to develop severe illness subtypes compared to patients with normal eosinophil levels. The underlying mechanisms linking eosinopenia with COVID-19 remain unclear at present. Previous studies have highlighted the role of cytokine storm as a central feature of severe COVID-19, which may contribute to eosinopenia (18, 19). In our study, patients with eosinopenia had higher levels of IL-10, which has been identified as a predictor for rapid diagnosis of COVID-19 patients at higher risk of disease deterioration (20).

Additionally, high levels of B-type natriuretic peptide (BNP), a marker of heart failure, were significantly higher in the eosinopenia group, which is consistent with previous reports suggesting its importance in treatment decision-making for COVID-19 patients (21). Furthermore, we found that laboratory biomarkers associated with the diagnosis and prognosis of COVID-19 patients, including glutamic oxaloacetic transaminase, serum creatinine, blood urea nitrogen, lactate dehydrogenase, creatine kinase, creatine kinase MB, myoglobin, and troponin I, were significantly higher in the eosinopenia group compared to the normal eosinophil group (22). COVID-19 patients with eosinopenia also required more pharmacotherapy and oxygen support, possibly due to their older age and higher prevalence of preexisting conditions. Cox regression analysis further supported eosinopenia as a valuable predictor of outcomes in Azvudine-treated COVID-19 patients, even after adjusting for potential confounders. Therefore, optimizing the management of patients with eosinopenia may help improve the prognosis of COVID-19 patients.

This study evaluated the differences in baseline characteristics, treatment, and outcomes between COVID-19 patients with eosinopenia and those with normal eosinophil levels. Most clinical features did not significantly differ between the two groups. However, decreased lymphocyte count was commonly observed in our cohort, which is consistent with previous studies (16). Our results indicated that COVID-19 patients with eosinopenia had higher levels of procalcitonin, which has recently been identified as a predictor of severity in hospitalized COVID-19 patients (21). A previous study suggested that high levels of B-type natriuretic peptide (BNP), a marker of heart failure, were significantly higher in the eosinopenia group, which is consistent with previous reports suggesting its importance in treatment decision-making for COVID-19 patients (22). Furthermore, we found that laboratory biomarkers associated with the diagnosis and prognosis of COVID-19 patients, including glutamic oxaloacetic transaminase, serum creatinine, blood urea nitrogen, lactate dehydrogenase, creatine kinase, creatine kinase MB, myoglobin, and troponin I, were significantly higher in the eosinopenia group compared to the normal eosinophil group (23). COVID-19 patients with eosinopenia also required more pharmacotherapy and oxygen support, possibly due to their older age and higher prevalence of preexisting conditions. Cox regression analysis further supported eosinopenia as a valuable predictor of outcomes in Azvudine-treated COVID-19 patients, even after adjusting for potential confounders. Therefore, optimizing the management of patients with eosinopenia may help improve the prognosis of COVID-19 patients.

## 5 Limitation

Several limitations should be acknowledged in this study. Firstly, the study was conducted in a single center, and further investigations are needed to determine whether our findings are consistent among other subgroups of COVID-19 patients. Secondly, as a retrospective study, the presence of selection bias or indication confusion cannot be completely ruled out, despite our efforts to adjust for confounding factors. Thirdly, we failed to evaluate the radiologic or pathological data of COVID-19 patients due to data unavailability. Fourthly, the effect of eosinopenia on vaccine effectiveness was not assessed in this study because we aim to assess the prognostic value of eosinopenia in Azvudine-treated COVID-19 patients. Lastly, the underlying mechanisms of eosinopenia in COVID-19 prognosis were not fully characterized in this study. Future research is warranted to precisely elucidate the value of eosinopenia in Azvudine-treated COVID-19 patients.

## 6 Conclusion

The present study provides evidence that Azvudine-treated COVID-19 patients with eosinopenia experienced a poorer clinical outcome compared to those with normal eosinophil levels in real-world clinical practice. Therefore, patients with eosinopenia should take additional precautions to protect themselves against COVID-19, and increased surveillance and medical care are recommended, particularly for elderly patients and those with preexisting conditions.

## Data availability statement

The original contributions presented in the study are included in the article/**Supplementary Material**. Further inquiries can be directed to the corresponding authors.

## Ethics statement

The studies involving humans were approved by the Ethics Committee of Xiangya Hospital of Central South University (No. 202002024). The studies were conducted in accordance with the local legislation and institutional requirements. The ethics committee/institutional review board waived the requirement of written informed consent for participation from the participants or the participants' legal guardians/next of kin because this study is a retrospective study and a secondary utilization of medical records, and patient's privacy and personal information are protected.

## Author contributions

XW: Conceptualization, Formal analysis, Investigation, Methodology, Writing – original draft, Writing – review & editing, Data curation, Software. YD: Formal analysis, Investigation, Methodology, Writing – review & editing. QZ: Formal analysis, Investigation, Writing – review & editing, Data curation. GD: Formal analysis, Investigation, Writing – review & editing, Funding acquisition, Methodology, Resources, Writing – original draft. RW: Writing – review & editing, Conceptualization, Validation, Investigation. FZ: Formal analysis, Funding acquisition, Investigation, Methodology, Resources, Writing – original draft, Writing – review & editing, Conceptualization, Supervision.

## Supplementary material

Supplementary Figure 1 The distribution of AST **(A)**, CRP **(B)**, D-Dimer **(C)**, and IL-10 **(D)** in patients with eosinopenia and normal eosinophil using GraphPad Prism software (mean with SD); significance was tested by Mann–Whitney test.
