# Supplementary figures and images for: Association of eosinopenia with worsening prognosis in hospitalized Azvudine-treated COVID-19 patients: a retrospective cohort study
Source: Front Immunol. 2023 Dec 15;14:1320973. doi: 10.3389/fimmu.2023.1320973 (PMC10754985; doi:10.3389/fimmu.2023.1320973)

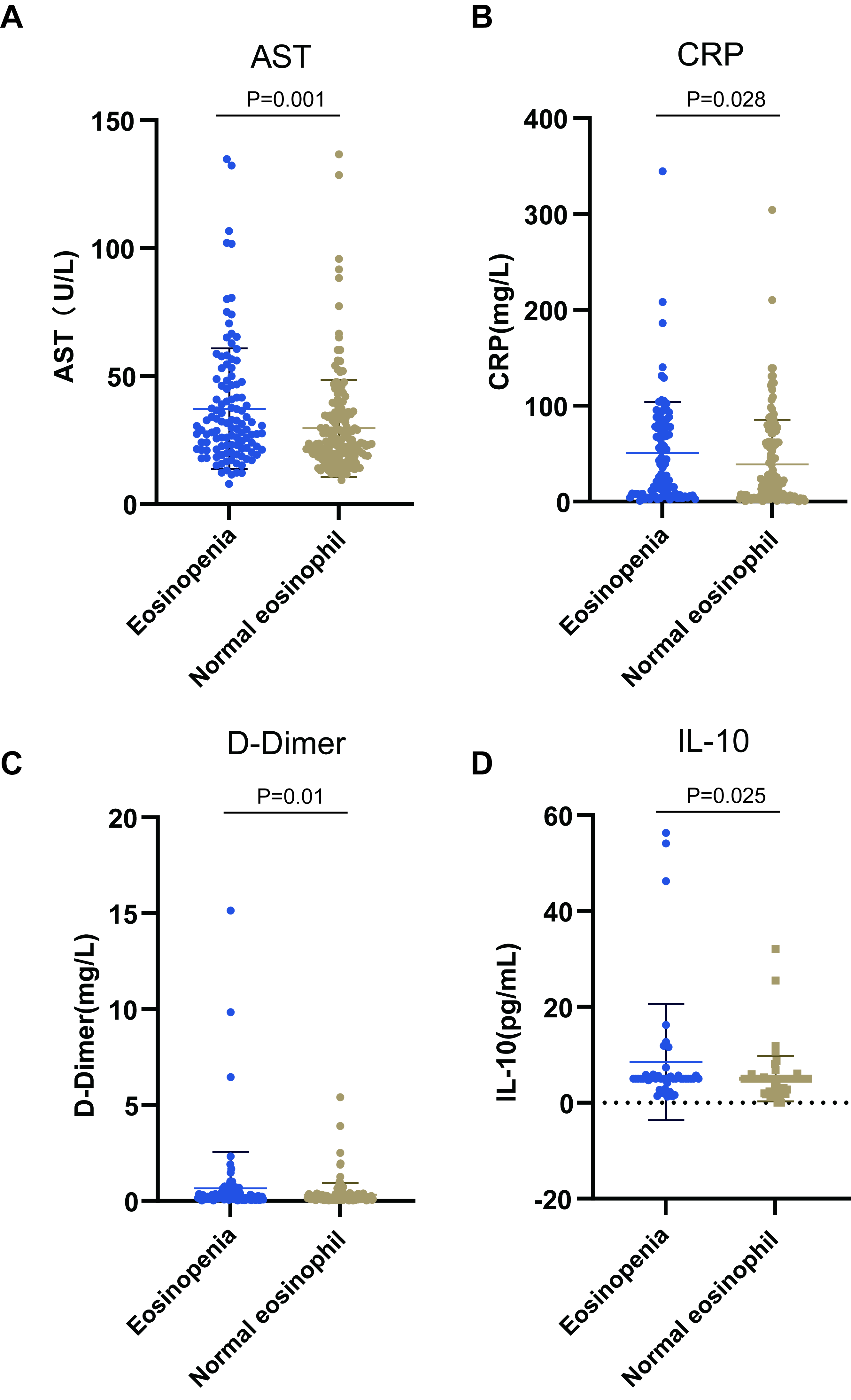

Supplement: Supplementary Figure 1 — The distribution of AST (A), CRP (B), D-Dimer (C), and IL-10 (D) in patients with eosinopenia and normal eosinophil using GraphPad Prism software (mean with SD); significance was tested by Mann–Whitney test. [file Image_1.tif]
